# Relationships Between Sleep Quality, Anxiety and Depression in University Students: Stable Trends over Time and a Pronounced Concern for Sleep Initiation

**DOI:** 10.3390/brainsci15111142

**Published:** 2025-10-24

**Authors:** Jonathan P. Whitehead, Caroline L. Horton

**Affiliations:** 1 DrEAMSLab, Department of Psychology, Lincoln Bishop University, Lincoln LN1 3DY, UK; 2 School of Natural Sciences, University of Lincoln, Lincoln LN6 7TS, UK

**Keywords:** sleep quality, sleep initiation, anxiety, mental health, sleep interventions

## Abstract

**Background/Objectives**: Relationships between sleep quality, anxiety and depression are well-documented across the lifespan. Here we investigated relationships between sleep, mental health and markers of obesity and cardiovascular health in Higher Education students (young adults, 18–28 years) using repeated cross-sectional sampling. **Methods**: Students (n = 486) participated at one of four timepoints across 2020–2023. The PSQI (sleep quality), GAD7 (anxiety) and PHQ8 (depression) were completed online. Measurements of obesity (Body Mass Index (BMI), body fat percent (BF%) and waist–hip ratio (WHR)) and cardiovascular function (heart rate (HR), diastolic and systolic blood pressure (DP and SP)) were determined. Changes over time, differences between sexes, and correlations between parameters were examined. **Results:** All measures were stable over the 4-year period. GAD7 (*p* < 0.0001) and PHQ8 (*p* = 0.0014) scores were significantly higher in females than males. There were significant, moderate to strong correlations between PSQI, GAD7 and PHQ8 scores for both sexes (r = 0.34–0.71). Only 18.1% of females and 23% of males reported both good quality sleep and no or low levels of anxiety and depression. Significant sex-specific differences were observed across markers of obesity and cardiovascular function (for BF%, WHR, HR and SP—all *p* ≤ 0.01), which showed weak to moderate correlations with sleep and mental health. Impaired sleep latency (C2) was identified as a potential key contributing factor. **Conclusions**: These observations provide evidence of multiple established, interlinked chronic challenges affecting sleep, mental and physical health in students. Identification of a key role for impaired sleep latency provides a foundation for targeted intervention, focusing upon improving sleep initiation, to improve mental health outcomes.

## 1. Introduction

Sleep is essential for physical and mental health, influencing functioning across both day and night [1]. Yet many individuals struggle to sleep well, and this can impair wellbeing even in the absence of a diagnosable disorder. Sleep health is multifaceted, encompassing depth, continuity, and subjective restfulness—not just duration [2]. 

The distinction between poor sleep and insomnia is critical. Insomnia refers to enduring sleep disturbances that meet clinical thresholds [3], with ≈8% of people meeting criteria for insomnia at any given time [4]. However, many individuals experience poor sleep without qualifying for a diagnosis. Indeed, UK data suggest ≈40% of people report sleep problems at any one time [5] with higher prevalence in populations with anxiety (≤50%) [6] and neurodiverse groups (≤80%) [7]. Moreover, subjective sleep quality is influenced by factors including perception, metacognition, and behavioural self-monitoring, and may diverge from physiological indicators [8,9]. Recent estimates show 64.9% of young adults experience sleep difficulties three or more times per week [10].

Associations between sleep quality and mental health are profound: Sleep disturbances are a hallmark of most mental health disorders, including anxiety, depression, trauma, and obsessive compulsions [11,12]. Globally, these associations are well documented, yet their mechanisms remain complex and bidirectional [11,13,14]. Sleep disruption has been shown to lead to increased anxiety and depression [15], psychotic symptoms [16], traumatic memory intrusions [17] and impaired emotional and cognitive outcomes [18]. While physiological markers may explain some pathways [19], mental health challenges are often multifactorial and comorbid. Promisingly, improving sleep may offer a holistic route to better coping [20], routine regulation [21] and emotional resilience.

Individual and environmental factors both contribute to poor sleep. For instance, anxious or neurotic personality traits may predispose individuals to sleep initiation difficulties [22], while environmental stressors—like studying in the bedroom or irregular schedules—can perpetuate poor sleep. These align with Spielman’s model of insomnia, which accounts for predisposing, precipitating and perpetuating factors [23]. 

Young adults, particularly University students, typically demonstrate poor sleep quality [24], often facing nutrition [25], exercise [26] and environmental and behavioural challenges: living independently for the first time, experiencing relative poverty [20], navigating inconsistent routines, noisy environments and suboptimal sleep settings. University students often experience additional pressures including academic demands, social transitions, and financial stress [27]. These factors can lead to late-night activity, alcohol use, fast food consumption and disrupted circadian rhythms [27]. For instance, in Dutch university students, poor sleep quality was independently associated with evening chronotype, depressive symptoms, smoking, and reduced emotion regulation [24]. Such patterns may perpetuate poor sleep and mental health in a cyclical fashion and support the development of targeted interventions [28].

In Chinese university students, adequate sleep duration and quality were associated with lower anxiety and depression [26]. However, a systematic review found that childhood sleep problems predicted later depression, while childhood anxiety and depression did not predict future sleep issues [29]. Anxiety was linked to excessive daytime sleepiness, whereas depression was not—highlighting distinct pathways and reinforcing that sleep quality is not synonymous with fatigue. In postgraduate students, those reporting restless sleep were over four times more likely to experience moderate to severe anxiety [30]. These findings suggest that specific facets of sleep—such as sleep latency—may be particularly relevant.

An additional predisposing factor is (biological) sex. National UK trends show rising rates of anxiety, depression, and stress in 16–24-year-olds from 2000 to 2020, with females having higher rates than males [19,21], likewise in China [31]. Behavioural moderators also differ by sex; for example, excessive daily internet use was a risk factor for low sleep duration in both young females and males, but it is a risk factor for anxiety in young females only [32]. Hence, investigating sex differences in sleep quality and the relationships with mental health may provide useful insights.

The COVID-19 pandemic introduced further complexity. For example, a study of young US adults (≈61% students) surveyed during the first two months of the pandemic reported high rates of sleep problems that were associated with worsening mental health, including symptoms of depression and anxiety [33]. Among students in Saudi Arabia, extremely high rates of depression and anxiety were linked to perceived pandemic impact [34]. 

These consistent trends are indicative of a complex behavioural and environmental profile that may exacerbate both poor sleep and mental health outcomes. Understanding sleep in university students is critical—not only for identifying symptom pathways but also for informing potential interventions.

Locally, anxiety is the third highest disease burden in people under 18 [32], and depressive disorders rank second in young adults. Lincolnshire Health Intelligence Hub estimates that 15.8% of adults have a recognised mental health disorder, yet university data suggest rates may be as high as 60% among students. 

In the present study, we report on data collected post-pandemic across four years, using the Pittsburgh Sleep Quality Index (PSQI) [11] rather than polysomnography to afford population-level comparisons due to its accessibility and ecological relevance [2]. The PSQI has been validated across diverse populations and regions. Meta-analyses show poor sleep quality in approximately half of older adults globally, with prevalence ranging from 96% in Malaysia, 27% across Africa, and 14% in China [35]. Sleep quality is also impaired in high-stress professions such as military personnel (69%) and firefighters (30%) [36,37]. Collectively, this demonstrates the PSQI’s sensitivity to detect sleep quality differences and changes over time. 

While observational, our repeated cross-sectional study design allows us to examine stability or change over time, without attributing effects directly to the pandemic. We asked young adults (18–28 years) enrolled at a UK university to complete online surveys assessing sleep quality, anxiety, depression, and physical health indicators. This process was repeated annually with independent samples across four years (2020–2023), beginning during the COVID-19 pandemic. Our aim was to explore general patterns of sleep quality behaviours in relation to mental and physical health symptoms and biomarkers, and to examine whether these relationships remained stable over time and differed by sex.

## 2. Materials and Methods

We wished to explore the sleep quality profile of University students in relation to wider health indices, and hypothesised the following: (1) Females would have poorer sleep quality, anxiety and depression than males; (2) correlations between sleep quality and anxiety and depression would be stronger for females than males; and (3) university students would demonstrate poor sleep quality (at least 40–60%, in line with [24] and [38]). Although we did not formulate specific predictions about the nature of sleep quality in this population as measured by the PSQI components, we wished to (4) explore associations between PSQI components, anxiety and depression across males and females, to help identify the profile of sleep quality in young adults. 

### 2.1. Study Ethics, Framework and Cohort Details

The overarching repeated cross-sectional study entitled “An Investigation into Associations between Student Lifestyle and Health and Wellbeing” was performed in accordance with the Declaration of Helsinki, and ethics approval was received from the School of Life Sciences ethics review committee (2020) and University of Lincoln ethics review committee (2021–2023: UoL2021_7296). Participants were recruited opportunistically by word of mouth, whereby those meeting eligibility criteria (namely, being a young adult university student at the participating university) were invited to take part by the student investigators, as outlined below. All participants provided informed (electronic) consent for their involvement in the overarching study, for personal data to be stored in accordance with the General Data Protection Regulation (GDPR), and for the prospective dissemination of the overarching study outcomes to be published in an anonymised format at the start of the survey (see Section 2.2). Participants received no remuneration for their involvement.

The overarching study was performed by a team of student investigators (typically around 30 per year). Each student investigator was supported and guided by an academic investigator to (i) develop their own research question under the umbrella of the overarching study, (ii) recruit up to five participants, and (iii) analyse the data for that year and report outcomes in a third-year dissertation. This paper presents an analysis of the relevant data collected as part of the overarching study across the years 2020–2023.

The final cohort comprised students enrolled at the University of Lincoln at the time of participation (n = 483, 296 ♀ and 187 ♂; median age 20 years). 

Data was collected from late November to mid-December across four consecutive years (2020 to 2023), using independent samples each year to allow sleep quality and associated health trends to be compared over time.

### 2.2. Survey—Self-Reported Sleep Quality, Anxiety and Depression

Participants completed an online survey that included an electronic consent form followed by a series of validated and non-validated questionnaires designed to explore aspects of student health and wellbeing. Participants were not required to complete all sections of the survey, which included validated instruments (PSQI, GAD-7, PHQ-8), and the order of items varied slightly across survey years. Missing responses may reflect participant fatigue, disengagement, or discomfort with specific questions. Rather than excluding partially completed surveys—which could introduce selection bias and disregard participant effort—we retained all available data for each measure.

The Pittsburgh Sleep Quality Index (PSQI) [11] is a popular, multi-factor self-report measure of sleep behaviours. It comprises 19 core self-rated items plus five additional items rated by a bed partner or roommate (if available), though only the self-rated items contribute to the scoring. Only the core items were included in the present study. These items are grouped into seven component scores: 1—Subjective sleep quality, 2—Sleep latency, 3—Sleep duration, 4—Habitual sleep efficiency, 5—Sleep disturbances, 6—Use of sleeping medication, 7—Daytime dysfunction. Each component is scored on a 0–3 scale, yielding a global score ranging from 0 to 21, with higher scores indicating poorer sleep quality. A global PSQI score > 5 is typically used to distinguish between good and poor sleepers. The PSQI differentiates between subjectively rated sleep quantity (components 3 and 4) from sleep quality (component 1), and sleep initiation (component 2, namely the time it takes to fall asleep) from sleep maintenance (component 5, namely the number of sleep disturbances and interruptions). The daytime dysfunction measurement (component 7) reflects the impact that sleep can have on waking function.

Although insomnia measures were collected in the first two waves, we focused on the PSQI due to its strong correlation with the Insomnia Severity Index [39] and its suitability for non-clinical populations.

The GAD-7 is a brief, 7-item self-report scale developed to identify probable cases of Generalized Anxiety Disorder (GAD) [40]. Each of its 7 items is scored on a 0 to 3 scale. The total GAD-7 score ranges from 0 to 21, with scores ≥5, 10, and 15 representing mild, moderate, and severe levels of anxiety, respectively.

The PHQ-8 (Patient Health Questionnaire-8) [41] is a self-report measure used to assess depression symptom severity. It comprises 8 items, which include all items from the PHQ-9 except for the one related to suicidal ideation (Item 9). Each item is scored on a 0 to 3 scale, yielding an overall PHQ-8 score ranging from 0 to 24. Overall scores of ≥5, 10, 15 and 20 represent mild, moderate, moderately severe and severe levels of depression, respectively.

PSQI, GAD7 and PHQ8 questionnaires were completed through an online survey, and responses were used to estimate sleep quality and levels of anxiety and depression. Other data collected via the survey included sex, age and ethnicity, and for participants in the 2020 cohort, measurements of height, weight, waist and hip (see below).

### 2.3. Anthropometric and Cardiovascular Measurements

Due to the impact of COVID-19 in 2020, participants were provided with guidance in the online survey on how to take height (m), weight (kg), waist (cm) and hip (cm) measurements with the aid of a housemate, or independently, and report them via the survey. In subsequent years (2021–2023), these anthropometric parameters were measured by trained student investigators when participants attended a scheduled appointment at the University of Lincoln. Cardiovascular parameters, including resting heart rate (HR) and diastolic and systolic blood pressure (DP and SP), were measured at the same appointment. All measurements were performed three times, and the mean value was recorded and used in subsequent analyses. Measurements across years 2021–2023 were performed using a Seca 217 stadiometer for height (Seca GmbH & Co. KG, Hamburg, Germany), Tanita BF-350 body composition scales for weight and body fat % (Tanita Corporation, Tokyo, Japan), a Seca fabric tape measure (waist and hip) and an Omron M6 comfort blood pressure monitor for HR, DP and SP (Omron Healthcare Co., Ltd., Kyoto, Japan).

### 2.4. Statistical Analysis

Data were collated using Microsoft Excel, and statistical analysis and visualisation were performed using GraphPad Prism v 10.5 (GraphPad Software LLC, San Diego, CA, USA). Statistical tests were selected based on the distributional properties of the data. Normality was assessed using the Anderson–Darling test, and appropriate parametric or non-parametric tests were applied accordingly. For comparisons between two groups, we used unpaired *t*-tests or Mann–Whitney U tests. For multiple group comparisons, we applied one-way ANOVA followed by Tukey’s post hoc test (parametric) or the Friedman test followed by Dunn’s multiple comparisons (non-parametric). Correlations were assessed using Pearson’s r (parametric) or Spearman’s rho (non-parametric). Details of specific statistical tests are provided in the Table header or Figure legend and relevant text. Typically, where data were normally distributed, means ± SD were reported, while median ± min/max or 95% confidence intervals (CIs) or inter-quartile range (IQR) were reported in all other cases.

## 3. Results

The cohort (n = 483, 61% female) predominantly identified as White (85%), with other ethnicities including Asian (5.4%), Black (4.8%), Mixed (2.1%), and Indian (1.2%). Additional backgrounds—each representing less than 1%—included Chinese, Pakistani, Arab, and Middle Eastern. Table 1 provides an overview of all the data described in more detail below.

**Figure 1:**
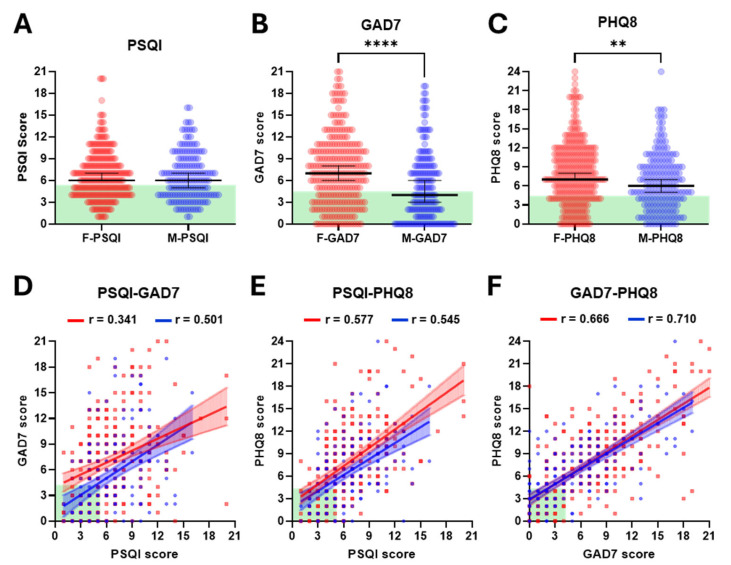
**Self-reported sleep quality, anxiety and depression scores and correlations in female and male students.** Panels (**A**–**C**) show PSQI (sleep quality, n = 273 ♀ and 171 ♂), GAD7 (anxiety, n = 291 ♀ and 182 ♂) and PHQ8 (depression, n = 290 ♀ and 183 ♂) scores for individual female (red) and male (blue) students and median ± 95% CIs (black lines). Statistical analysis was performed using Mann–Whitney tests (** *p* < 0.01, **** *p* < 0.0001). The green background depicts the ideal to adequate range for each score. Panels (**D**–**F**) show correlations between PSQI—GAD7 (n = 268 ♀ and 168 ♂), PSQI—PHQ8 (n = 267 ♀ and 169 ♂) and GAD7—PHQ8 (n = 289 ♀ and 182 ♂) scores with individual female (red) and male (blue) scores, lines of simple linear regression ± 95% CIs (all *p* < 0.0001) and Spearman correlation values (r). The green background depicts the ideal to adequate range for each score.

PSQI scores were comparable between females and males, both showing a median of 6 (mean ± SD; 6.8 ± 3.4 vs. 6.6 ± 3.3) (Figure 1A and Figure S1A). The majority of female (59%) and male (57%) participants had a PSQI score of 6 or higher, indicative of poor-quality sleep in most of the cohort. In contrast to the similarity between PSQI scores for females and males, females scored significantly higher than males in both GAD7 and PHQ8. For GAD7 scores, the median for females was almost twice that for males (7 vs. 4, *p* < 0.0001: mean ± SD; 7.3 ± 4.9 vs. 5.4 ± 4.8) (Figure 1B and Figure S1B), with 68% of females having scores of 5 or higher, indicative of moderate to severe anxiety, compared with only 49% of males. Although the difference between the sexes was more moderate for PHQ8 scores, a similar pattern was observed (median 7 vs. 6, *p* = 0.0014: mean ± SD; 8.0 ± 5.0 vs. 6.6 ± 4.5) (Figure 1C and Figure S1C). PHQ8 scores of 5 or higher, indicative of moderate to severe depression, were reported in 73% of females and 64% males. Further analysis revealed that only 18.1% of female and 23% of male participants had scores indicative of good quality sleep and no or low levels of anxiety and depression. We also performed linear regression analysis to investigate potential correlations between PSQI, GAD7 and PHQ8 scores, analysing females and males separately, considering the differences described above. In all cases, significant correlations were found (all *p* < 0.0001; Figure 1D–F). Although qualitatively different, there were no significant differences (all *p* > 0.1) between the slopes of the regression lines for females cf. males for PSQI vs. GAD7 (0.47 cf. Apol0.65), PSQI vs. PHQ8 (0.81 cf. 0.71) or GAD7 vs. PHQ8 (0.71 cf. 0.68) (Figure 1D–F). Notwithstanding, these findings collectively reaffirm the strong interactions between sleep, anxiety and depression and serve to highlight the low proportion of students who report no challenges across all three parameters.

**Figure 2:**
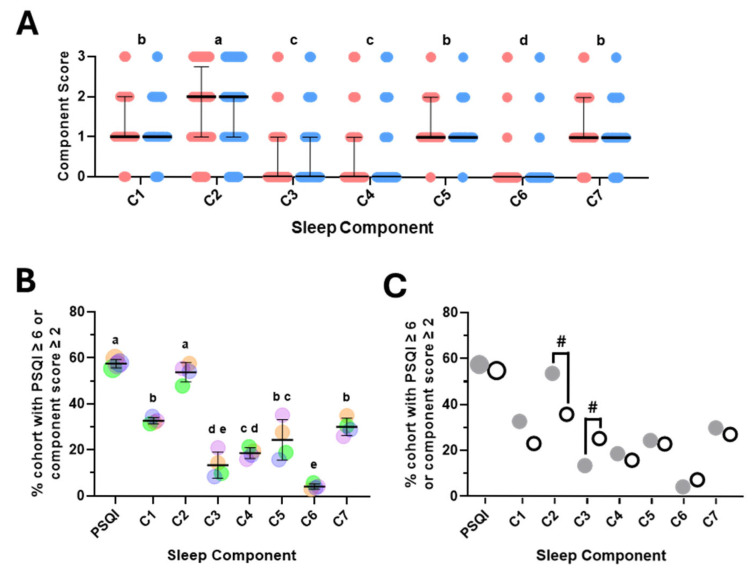
**Contribution, distribution and comparison of sleep component scores in students.** (**A**) Distribution and median ± IQR for sleep component scores, C1–C7, in female (red, n = 273) and male (blue, n = 171) students. Statistical analysis was performed on the combined female and male scores for each sleep component using the Friedman test followed by Dunn’s multiple comparisons test. Components that share the same letter (a–d) were not significantly different. (**B**) Percentage (%) of students with overall PSQI score or sleep component score greater than the threshold (PSQI ≥ 6, C ≥ 2) are shown for individual years 2020 (orange, n = 96), 2021 (green, n = 121), 2022 (lilac, n = 119) and 2023 (blue, n = 107). Combined means ± SD are presented as black lines. Statistical analysis was performed using ordinary one-way ANOVA followed by Tukey’s multiple comparisons. Factors with the same letter (a–e) were not significantly different. (**C**) Percentage (%) of students with overall PSQI score or sleep component score greater than the threshold (PSQI ≥ 6, C ≥ 2) are shown for this cohort (UoL, grey circles) and a comparable student cohort from the UAE (open circles, n = 379—see [38]). ^#^ *p* < 0.0001 denotes significant differences between median values for C2 and C3 for UoL cf UAE using Kruskal–Wallis test followed by Dunn’s multiple comparisons.

The overall PSQI score is a composite score made up of seven component scores, each representing a discrete aspect of sleep/wake behaviour [11]. To investigate whether one or more of the component scores may have contributed significantly more to the overall PSQI score, we first analysed the distribution of the component scores. This approach revealed there were no significant differences between females and males and that the sleep latency score (C2) was significantly higher than all other scores (Figure 2A). Scores for subjective sleep quality (C1), sleep disturbances (C5) and daytime dysfunction (C7) were significantly higher than sleep duration (C3) and habitual sleep efficiency (C4), whilst use of sleep medications (C6) scored significantly lower than all others (Figure 2A). To explore this further, we determined the percent of participants who had an overall PSQI score, or an individual component score deemed less than ideal or inadequate (≥6 for PSQI and ≥2 for each component score). This analysis revealed comparable findings to those above, whilst also highlighting the similarity between the percentage of participants with inadequate PSQI and C2 scores (Figure 2B). To examine this in a second independent cohort, we took advantage of a recent study that reported overall PSQI and sleep component scores in a relatively comparable student cohort in the United Arab Emirates (UAE) (n = 379, 64% female, aged 18–30) [38]. Interestingly, this revealed strong similarities (PSQI and C4, C5, C6 and C7) as well as potentially intriguing differences (C2 and C3) (Figure 2C). Whilst sleep latency score (C2) was the most prevalent inadequate sleep component across both cohorts, it was considerably lower in the UAE cohort (35.8% vs. 53.6%). Consistent with this, the median C2 score was significantly lower in the UAE cohort (1 vs. 2, *p* < 0.0001—one-way ANOVA followed by post hoc Kruskal–Wallis test). In contrast, the percent of participants with an inadequate C3 score and the median C3 score were both higher in the UAE cohort (25.3% vs. 13.5% and 1 vs. 0, *p* < 0.0001—Kruskal–Wallis test followed by Dunn’s multiple comparisons test).

**Figure 3:**
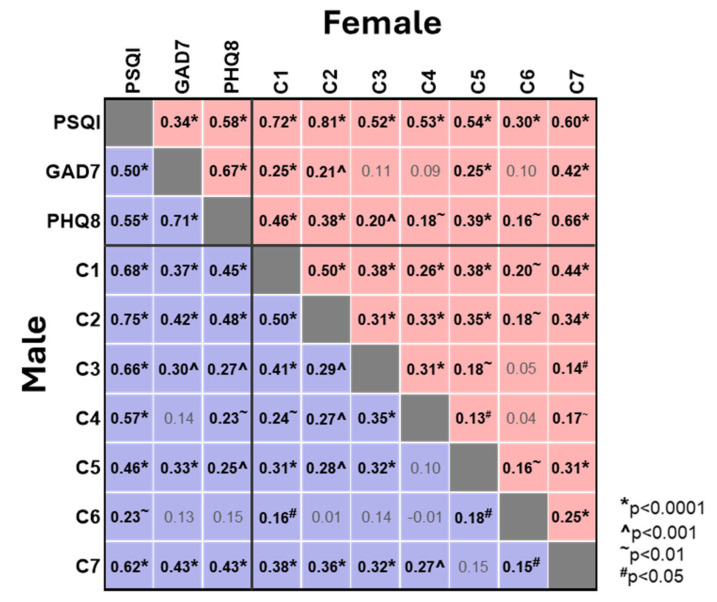
**Correlation matrix between sleep quality, anxiety, depression and sleep component scores in female and male students**. Correlations for females (red) and males (blue) are presented in the upper right and lower left of the grid, respectively. Spearman correlation values (r) are shown. Where significant, correlations are highlighted in bold with *p* values indicated (* *p* < 0.0001, ^ *p* < 0.001, ^~^ *p* < 0.01, ^#^ *p* < 0.05).

We next investigated the correlations between the individual sleep component scores and the overall PSQI score and the GAD7 and PHQ8 scores (Figure 3). As expected, and consistent with the above, all sleep component scores showed a positive correlation with the overall PSQI score, with the strongest correlation observed for sleep latency (C2, Spearman’s r values 0.81 and 0.75 for ♀ and ♂) and the weakest correlation for use of sleep medication (C6, 0.30 and 0.23 for ♀ and ♂). Perhaps more importantly, there were several moderate correlations between the individual sleep component scores and the GAD7 and PHQ8 scores (Figure 3). In females the strongest correlation with both GAD7 and PHQ8 was daytime dysfunction (C7, 0.42 and 0.46), whilst sleep latency (C2, 0.21 and 0.38) showed similar correlations to subjective sleep quality (C1, 0.25 and 0.46) and sleep disturbances (C5, 0.25 and 0.39). Whilst the pattern in males was similar, sleep latency (C2) showed the strongest correlation to PHQ8 (0.48) and was comparable with daytime dysfunction (C7) in its correlation with GAD7 (C2 vs. C7, 0.42 vs. 0.43) (Figure 3). Challenges with sleep latency, sleep quality and sleep disturbances are deeply intertwined with resultant daytime dysfunction, reinforcing the association between poor sleep and heightened anxiety and depression.

**Figure 4:**
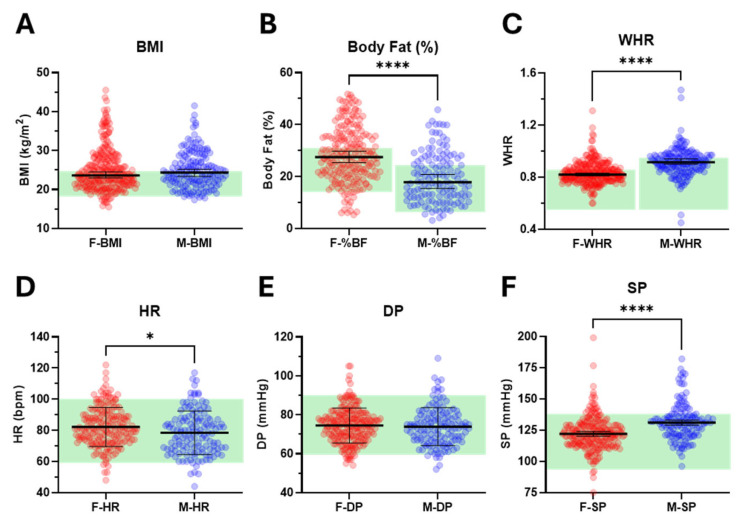
**Measures of obesity and cardiovascular status in female and male students.** Panels show (**A**) body mass index (BMI, n = 260 ♀ and 162 ♂), (**B**) body fat percent (BF%, n = 214 ♀ and 135 ♂), (**C**) waist–hip ratio (WHR, n = 268 ♀ and 170 ♂), (**D**) heart rate (HR, n = 194 ♀ and 136 ♂), (**E**) diastolic pressure (DP, n = 216 ♀ and 136 ♂) and (**F**) systolic pressure (SP, n = 217 ♀ and 136 ♂) for individual female (red) and male (blue) students. Median ± 95% CIs are presented in panels (**A**–**C**,**F**), whilst means ± SD are presented in panels (**D**,**E**) (black lines). Statistical analysis was performed using Mann–Whitney tests for data presented in panels (**A**–**C**,**F**) and unpaired *t*-tests for data presented in panels (**D**,**E**). Significant differences between females and males are indicated (* *p* < 0.02, **** *p* < 0.0001). The ideal to adequate range for each measurement is highlighted by the green background region.

We examined additional features related to sleep and both mental and physical health, focusing on commonly used measures of obesity (BMI, BF%, and WHR) and cardiovascular status (HR, DP, and SP). While median BMI was comparable between females and males (23.6 vs. 24.4 kg/m^2^), females exhibited significantly higher median BF% (27.5 vs. 17.8%) and lower median WHR (0.82 vs. 0.92) (Figure 4A–C, Table 1 and Supplementary Figure S2A–C). Mean resting HR was significantly higher in females compared with males (82 vs. 79 bpm) (Figure 4D, Table 1 and Supplementary Figure S2D). Although mean DP (75 vs. 74 mmHg) was similar between the sexes, median SP was significantly lower in females (122 vs. 131 mmHg) (Figure 4E,F, Table 1 and Supplementary Figure S2E,F).

The percentage of the cohort with elevated measures of obesity was ≈40% (36.7 ± 3.4 and 44.0 ± 12.3 (mean ± SD) for ♀ and ♂), whilst only ≈9% showed elevated markers of cardiovascular function (7.1 ± 2.1 and 11.5 ± 7.9 for ♀ and ♂). Striking sex-specific differences were apparent for WHR and SP, with a much higher proportion of males than females with elevated WHR and SP (56.5 vs. 33.2% and 20.6 vs. 8.3%—see Supplementary Table S1).

**Figure 5:**
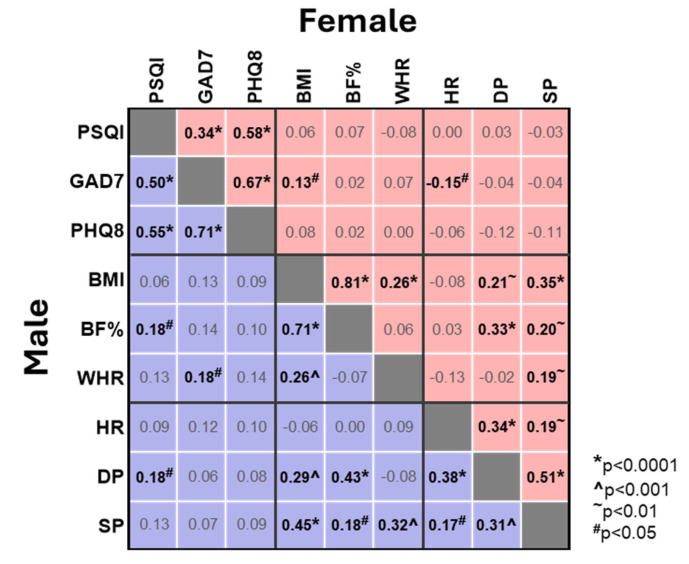
**Correlation matrix between sleep quality, anxiety, depression and measures of obesity and cardiovascular status in female and male students.** Correlations for females (red) and males (blue) are presented in the upper right and lower left of the grid, respectively. Spearman correlation values (r) are shown. Where significant, correlations are highlighted in bold with *p* values indicated (* *p* < 0.0001, ^ *p* < 0.001, ^~^ *p* < 0.01, ^#^ *p* < 0.05).

We then assessed correlations within and across categories, confirming expected strong associations between distinct measures of obesity, such as BMI and BF% (Spearman’s r = 0.81 and 0.71 for ♀ and ♂), and between obesity measures and cardiovascular status, such as BMI and SP (Spearman’s r = 0.35 and 0.45 for ♀ and ♂) (Figure 5). In contrast, while some correlations between measures of obesity or cardiovascular status and sleep or anxiety were identified, these were weak (Spearman’s r = 0.13 to 0.18) and of low significance (*p* < 0.05) (Figure 5).

## 4. Discussion

The aim of the present study was to investigate and characterise sleep quality in young adult undergraduate students (age 18–28 years), to explore trends in independent samples over time, to explore possible associations with mental health measures (anxiety and depression), as well as wider indicators of physical health.

### 4.1. Sleep Quality Is Related to Mental Health

Sleep quality was shown to be consistently poor, and in line with prior explorations of sleep health in student populations [38,42,43]. Furthermore, sleep latency (C2) appears to be a major contributor to poor sleep quality, which is also consistent with previous reports [42,43]. The term “insomnia” is frequently used to refer to poor sleep, which can be characterised by difficulty in maintaining sleep as well as initiating it. However, our observational data suggest that efforts should be directed towards trying to understand and potentially improve sleep initiation by raising awareness of how to “switch off” in order to improve sleep and related outcomes. Evidence suggests this will improve student health and wellbeing [44].

In this sample of young adults demonstrating a range of challenging physical and mental health indices, sleep quality was poor as measured by overall subjective sleep quality, sleep latency (sleep initiation) and the impact of these on daytime functioning. This serves as a stark reminder that sleep impacts waking function, both cognitively and in relation to mental health, and should, therefore, be conceptualised holistically. Importantly, very few participants (approximately 20% overall) reported both good quality sleep and mental health outcomes (i.e., low levels of anxiety and depression). Young adults studying at university in our sample demonstrated very high associations between sleep quality and mental health outcomes. Their health was poor and in need of support for improvement.

Consequently, although healthy sleep likely drives other systematic health behaviours such as eating [24,25], exercise [26] and immune function [25], public health initiatives to improve sleep and/or increase awareness of the importance of sleeping well have historically been limited (e.g., [4]) and our assessments of these in contemporary public health agenda in the UK and beyond indicate that very little has changed to prioritise sleep health at the population level.

This is unfortunate for many reasons, not least that improving sleep can offer a means by which other physical and mental health issues could be lessened or prevented. Furthermore, specific groups who may characteristically struggle with their sleep quality and/or who could be vulnerable to poor health outcomes that their sleep affects are missing the opportunity to improve their sleep and, in turn, their other health behaviours.

The findings reported here demonstrated strong relationships between sleep quality and depression and anxiety, as well as consistency in the strength of these associations over time. We did not explore the specific mechanisms by which disordered sleep may impact poor mental health, though the observed trends clearly indicated that sleep latency, as a measure of sleep initiation (rather than maintenance), drove poor sleep quality and, therefore, likely, the relationships with anxiety and depression also. This trend has been evidenced elsewhere (e.g., [43]) in a sample of nursing and technical university students in Austria.

### 4.2. Sex Differences

Our data further suggest that the relationships between specific components of sleep quality and anxiety and depression differ between males and females. Specifically for females, daytime dysfunction was most strongly correlated with both anxiety and depression outcomes (out of all sleep quality components). Meanwhile, data from male participants indicated that sleep latency, or the time taken to fall asleep, was most strongly associated with depression, whilst both sleep latency and daytime dysfunction were similarly (strongly) related to anxiety. Poor daytime dysfunction scores may result from insufficient sleep or may reflect struggles to function due to mental health challenges. Due to the inextricable links between these, it is difficult to disentangle cause from effect as well as symptom from stressor.

These sex differences broadly reflect prior published evidence that is well defined (e.g., [21]). In the same way that relationships between poor sleep quality and mental health outcomes are inextricably linked, it can be very difficult to unpack whether any sex differences primarily reflect differences in sleep behaviours, or anxiety or depression. Our results illustrated that sleep quality parameters were comparable for males and females, but females had significantly higher rates of both depression and anxiety than males. As such, although both mental health measures were significantly correlated with sleep quality components, there remained substantial variance in the mental health indices that could not be explained by sleep quality. Although our data are merely observational, the findings may direct focus towards a range of mechanisms by which sleep impacts mental health and vice versa.

One study [44] demonstrated significant associations between insomnia severity and sexual orientation, but those relationships disappeared once depression and anxiety measures were controlled for, which in this case indicated that differences in mental health outcomes could account for much variance in sleep outcomes. Sexual orientation is not the same as sex, of course, so these trends could be further moderated by being female.

Whether the behavioural disturbances originate in poor mental health or poor sleep quality, it seems that challenges with sleep latency, sleep quality and sleep disturbances are deeply intertwined with resultant daytime dysfunction, reinforcing the association between poor sleep and heightened anxiety and depression.

### 4.3. Mechanisms of Action

Further real-world research is needed to understand the mechanisms by which difficulties with sleep initiation relate to anxiety and depression, most notably whether an inability to fall asleep leads to increased anxiety and depression through reducing sleep quantity, or whether anxiety and depression lead to an inability to switch off through, for instance, increased rumination [45]. Existing experimental evidence suggests a role for both actions, such that in practice, a cyclical relationship likely exists between disturbed sleep and disordered emotion regulation.

Rumination is a feature of both anxiety and depression, though more commonly with the latter. Experimental manipulations (e.g., [46]) demonstrate how persistent rumination can be easily cued by an unresolved personal goal, and further evidence demonstrates the effects of rumination on sleep quality [45], specifically such that pre-sleep arousal mediates relationships between stress and sleep quality in high ruminators (much more than low ruminators). This also implies that a high cognitive load, whether emotionally neutral or not, could influence sleep initiation. Coupled with the arousal from emotional concerns or anxieties, the conditions for transitioning from wakefulness to a restful, sleep state would be impeded.

Although much evidence, including the data presented in this paper, demonstrates correlations between poor sleep and mental health outcomes, rather than indicating causation, some experimental work has established increases in anxiety outcomes following sleep deprivation over a single night (e.g., [15]). Few studies have been able to track these behaviours over a longer, more real-world timeframe, but a large-scale population-based survey study in Norway [47] attempted this over two timepoints, eleven years apart. The outcomes demonstrated that insomnia symptoms at time 1 were related to anxiety, but not depression, symptoms at time 2. Relationships between disturbed sleep and both anxiety and depression outcomes were evident at the same timepoints, reinforcing the co-existence of these maladaptive behaviours. This lends some real-world support to the idea that highly disturbed sleep may increase the risk of anxiety, specifically.

Although depressive behaviours can develop in much the same ways as maladaptive sleep habits, a personal loss is often the origin of depression. Non-pharmacological treatments for depression, anxiety and insomnia frequently adopt cognitive-behavioural approaches, as a recognition of both the behavioural and individualised belief systems that may underpin those behaviours. Cognitive biases around sleep need or usefulness may lead university students to believe that staying up all night to study may be a productive approach. Wider cultural influences, such as student nights or having inconsistent work routines, may perpetuate poor sleep habits. This, in turn, could lead to a misalignment between sleep and work schedules, increasing the chances of university students sleeping later, at sub-optimal times of the night or day. Collectively, this hypothetical example acts as a further reminder that sleep quality is more multifaceted and complex than mere sleep duration (e.g., [2]), with healthy sleep occurring regularly, easily, and at appropriate times to ensure sufficient sleep depth and continuity.

### 4.4. Associations with Wider Physical Health Metrics

Although the main aims of the present study were to characterise the nature of sleep quality in young adults over time and to explore associations with mental health indices, a number of simple measures of obesity and markers of cardiovascular function were also included. As reported, there were limited associations between sleep, anxiety or depression and these parameters. Taken at face value, the variance in sleep quality, anxiety or depression in young adults was not accounted for by variance in obesity and cardiovascular health. Whilst the impact of sleep on physical health outcomes has been well-documented, differing aetiologies and timelines of progression are likely to contribute to these findings in this relatively young cohort. Consistent with this, it is noteworthy that the prevalence of obesity across the cohort was relatively high, at around 40%, whilst the incidence of elevated markers of cardiovascular status, most notably elevated diastolic and or systolic blood pressure, was relatively low, at only 9%. The causal relationship between obesity, hypertension and future cardiovascular risk in young adults is an area of increasing research interest [48].

### 4.5. Study Limitations

The reported data were collected over the period of the pandemic, demonstrating that sleep quality and mental health outcomes did not worsen in line with the pandemic. In fact, comparable data from the locality [49] also reported that mental health rates were slightly higher in 2019, pre-pandemic, relative to the immediate pandemic years, particularly in adolescents. Here, we have been able to observe trends from independent samples not only over the acute pandemic period, as in [33], but also over the subsequent years. We acknowledge that sampling repeated measures could have limited some of the variance within these data, but we wished to collect data from samples over time at the same point in their university studies. 

Although these outcomes reflect trends observed in other comparable datasets (see also [34,42,43]), the associations were observed in the data, with sleep quality and mental health measurements being self-reports. We are, therefore, cautious with any explanation of the potential impact (or not) of the COVID-19 pandemic, or indeed of the potential direction of causality between sleep and mental health outcomes, especially when a broad range of health indices were obtained.

Finally, although data were collected from university students who were young adults, some caution should be applied when considering the representativeness of university students of all young adults.

### 4.6. Future Directions

It seems pertinent to focus on enhancing sleep health for university students through supporting sleep initiation, given that extensive poor sleep quality was principally driven by poor (high) sleep latencies. Strategies to improve sleep have been shown to be most effective when including both a cognitive element and a structured programme for instigating a bedtime routine. Existing sleep-support mechanisms rely heavily upon CBTi (see [3]), and these can be highly effective, particularly for individuals with persistent insomnia. A systematic review and meta-analysis of interventions in university students indicated small effect sizes for the usefulness of sleep interventions on both improved sleep outcomes and anxiety [50], with greater effects for those with insomnia compared to those with less problematic sleep. However, as demonstrated here, university students may not meet the diagnostic threshold for insomnia yet still suffer with their sleep and associated health behaviours. Given the characteristic poor sleep quality reported in the present paper, there is hope that related interventions may be useful not only in alleviating some of the poor sleep directly, but also in boosting wellbeing even in the absence of an insomnia diagnosis, though more work is required to explore this [51].

As stated previously, our data demonstrated an extremely high correlation between Insomnia Severity Index scores and PSQI scores, indicating that poor sleep quality shares characteristics and variance with insomnia, without the diagnostic label. Qualitative work exploring the perceptions of and associations with the term “insomnia” would help to elucidate whether using the term more readily may be a help or a hindrance in allowing students to identify their sleep behaviours.

Nevertheless, sleep improvement interventions that emphasise individual responsibility may also implicitly apportion blame for poor sleep health, which is unethical given the range of cultural factors that exacerbate poor sleep that are beyond the control of the individual student (e.g., inconsistent schedules, poor living conditions and environments). Consequently, sector-wide sleep campaigns and sleep support strategies should be explored further.

Whilst the effectiveness of large-scale sleep improvement programmes is varied and the cognitive mechanisms of success are likewise mixed [51], supporting sleep health at scale is crucial to support individuals with their overall health, as well as their ability to learn as a student [52]. Relationships between sleep health and stress are profound in undergraduate students [53], which may reflect increased stress resulting from insomnia. Taken together, the imperative to try to improve sleep in university students is strong and may well bolster resilience to future mental ill-health. It is also important to consider treating anxiety, especially for those with generalised anxiety and not merely anxiety in relation to sleep health [54].

In the present paper, we have conceptualised undergraduate student sleep and associated mental health broadly, and the findings align with prior trends in support of this notion. However, there is, of course, heterogeneity within student populations, and the individual differences within those should not be dismissed. For instance, data collected from veterinary students showed extremely high levels of stress, insomnia severity, anxiety and depression relative to the [Austrian] general population [55]. However, these students worked on professional placements in high-stress environments and may, therefore, not be representative of other young adults studying less demanding subjects. Nevertheless, our data presented here, along with the wealth of data from comparable studies, indicate disturbed sleep and associated mental health challenges that require sector-wide intervention and support, which may be protective against future worsening health challenges.

## 5. Conclusions

These observations highlight multiple chronic interlinked challenges affecting sleep quality and overall mental and physical health in university students. A key role for impaired sleep latency was identified, providing a foundation for targeted interventions focused on improving sleep initiation and, in turn, mental health outcomes.

We explored sleep alongside a range of health behaviours over time, predicting and finding associations between poor sleep quality, depression and anxiety across the pandemic and post-pandemic periods. Consistent with prior evidence, sleep quality was consistently poor in this sample, mirroring wider trends in higher education students. Difficulties initiating sleep were a primary contributor that were closely linked to symptoms of depression and anxiety, and we wish to highlight the significance of sleep initiation challenges for young adults. Although the precise determinants of the difficulties with initiation need to be explored and understood further, this can be achieved by adopting experimental methods to delineate directions of causation, as well as implementing sleep improvement techniques. Further, such endeavours should shift the focus away from understanding sleep maintenance and towards readiness to sleep.

## Figures and Tables

**Table 1:** Summary of all measures across the whole cohort (all) and grouped by biological sex (female and male).

	n			Mean (±SD)			Median (min/max)			*p* Value
	All	F	M	AU	F	M	All	F	M	FvM
**Age**	483	296	187	**20.7** (±1.4)	**20.6** (±1.4)	**20.8** (±1.4)	**20** (18/28)	**20** (18/28)	**20** (18/28)	0.1713
**PSQI**	444	273	171	**6.7** (±3.3)	**6.8** (±3.4)	**6.6** (±3.3)	**6** (1/20)	**6** (1/20)	**6** (1/16)	0.6079
**GAD7**	473	291	182	**6.6** (±4.9)	**7.3** (±4.9)	**5.4** (±4.8)	**6** (0/21)	**7** (0/21)	**4** (0/19)	**<0.0001**
**PHQ8**	473	290	183	**74** (±4.9)	**8.0** (±5.0)	**6.6** (±4.5)	**7** (0/24)	**7** (0/24)	**6** (0/24)	**0.0014**
**BMI**	422	260	162	**25.0** (±5.2)	**25.1** (±5.5)	**25.0** (±4.7)	**23.9** (15.5/45.5)	**23.6** (15.5/45.5)	**24.4** (17.3/41.5)	0.5290
**BF%**	349	214	135	**25.0** (±11.0)	**28.4** (±10.6)	**19.6** (±9.5)	**24.2** (3.1/51.8)	**27.5** (5.3/51.8)	**17.8** (3.1/45.7)	**<0.0001**
**WHR**	438	268	170	**0.86** (±0.11)	**0.83** (±0.09)	**0.92** (±0.11)	**0.86** (0.31/1.47)	**0.82** (0.6/1.31)	**0.92** (0.45/1.47)	**<0.0001**
HR *	330	194	136	**81** (±13)	**82** (±13)	**79** (±14)	**80** (44/122)	**81** (48/122)	**78** (44/117)	**0.0129 ^t^**
DP *	352	216	136	**74** (±9)	**75** (±9)	**74** (±10)	**74** (52/109)	**75** (54/105)	**74** (52/109)	0.5286 ^t^
**SP**	353	217	136	**126** (±15)	**123** (±14)	**132** (±15)	**125** (75/199)	**122** (75/199)	**131** (96/182)	**<0.0001**

Differences between females and males (*p* values) were calculated using the Mann–Whitney test, except for HR and DP, which were normally distributed * and calculated using an unpaired Student’s *t*-test, t Those *p* values denoting significant differences between female (F) and male (M) are shown in bold.

## Data Availability

The original contributions presented in this study are included in the article/Supplementary Material. Further inquiries can be directed to the corresponding authors.

## Supplementary Materials

The following supporting information can be downloaded at: https://www.mdpi.com/article/10.3390/brainsci15111142/s1, Figure S1: Self-reported sleep quality, anxiety and depression scores and in female and male students grouped by year; Figure S2: Measures of obesity and cardiovascular status in female and male students grouped by year; Table S1: Summary of elevated measures of obesity and cardiovascular function.

## Abbreviations

The following abbreviations are used in this manuscript:

PSQI	Pittsburgh Sleep Quality Index
GAD7	General Anxiety Disorder 7th edition (measure of anxiety)
PMQ8	Patient Medical Questionnaire 8 (measure of depression)
