# Supplementary material for: Relationships Between Sleep Quality, Anxiety and Depression in University Students: Stable Trends over Time and a Pronounced Concern for Sleep Initiation
Source: Brain Sci. 2025 Oct 24;15(11):1142. doi: 10.3390/brainsci15111142 (PMC12650024; doi:10.3390/brainsci15111142)
Supplement: Supplementary file 1 [file brainsci-15-01142-s001.zip › brainsci-3877117-supplementary.pdf]

Supplementary Materials

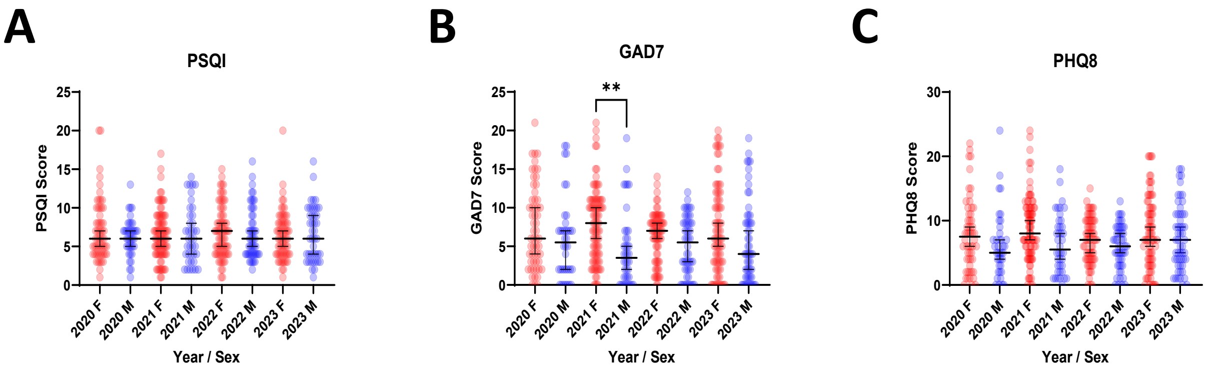

Supplementary Figure S1. Self-reported sleep quality, anxiety and depression scores and in female and male students grouped by year. Panels show (A) PSQI (sleep quality, 2020 to 2023, n=58, 82, 69 & 64 ♀ and 39, 39, 50 & 43 ♂), (B) GAD7 (anxiety, 2020 to 2023, n=57, 88, 69 & 77 ♀ and 38, 40, 50 & 54 ♂) and (C) PHQ8 (depression, n=56, 88, 69 & 77 ♀ and 39, 40, 50 & 54 ♂) scores for individual female (red) and male (blue) students and median ± 95% CIs (black lines). Kruskal-Wallis test followed by Dunn's multiple comparisons test to identify significant differences between females vs males in the same year and females or males across different years (**p=0.0019).

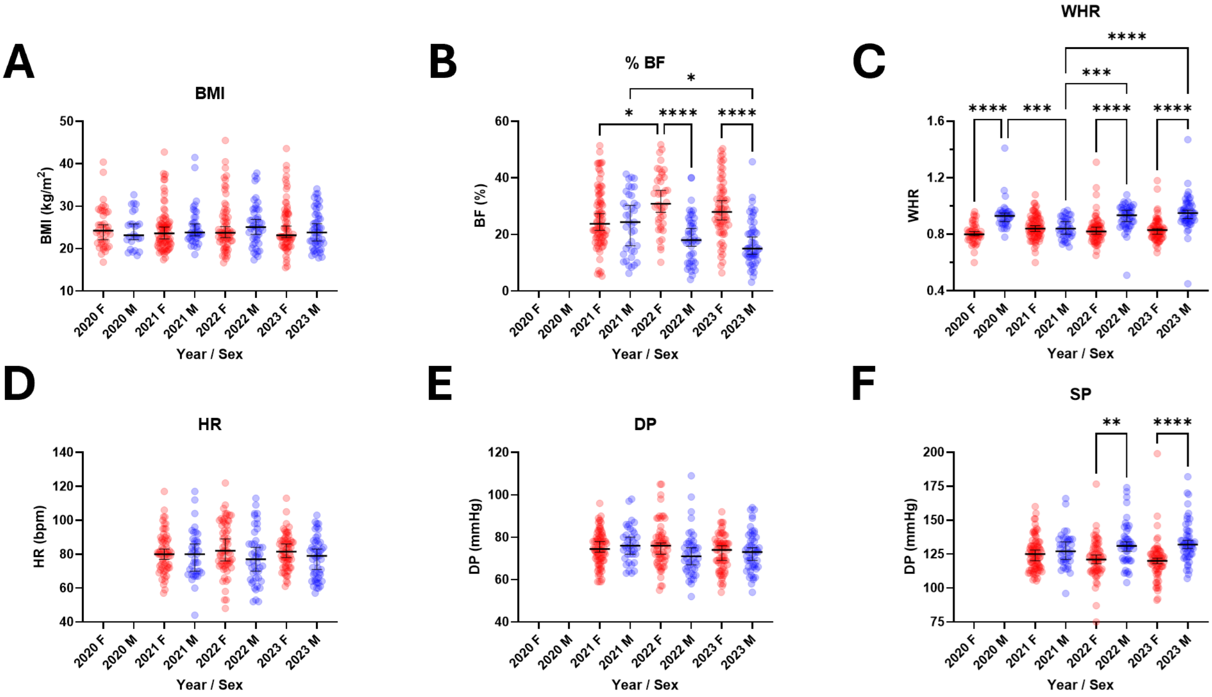

Supplementary Figure S2. Measures of obesity and cardiovascular status in female and male students grouped by year. Panels show **(A)** BMI (n=42, 84, 66 & 68 ♀ and 26, 39, 46 & 51 ♂), **(B)** BF% (n=0, 84, 62 & 68 ♀ and 0, 39, 45 & 51 ♂), **(C)** WHR, n=49, 85, 66 & 68 ♀ and 34, 39, 46 & 51 ♂), **(D)** heart rate (HR, n=0, 63, 65 & 66 ♀ and 0, 39, 46 & 51 ♂), **(E)** diastolic pressure (DP, n=0, 84, 65 & 67 ♀ and 0, 39, 46 & 51 ♂) and **(F)** systolic pressure (SP, n=0, 85, 65 & 67 ♀ and 0, 39, 46 & 51 ♂) for individual female (red) and male (blue) students. Median ± 95% CIs are presented (black lines). Statistical analysis was performed using Kruskal-Wallis test followed by Dunn's multiple comparisons test to identify significant differences between females vs males in the same year and

females or males across the different years (*p<0.05, **p<0.01, ***p<0.001, ****p<0.0001). No data for BF%, HR, DP or SP were collected in 2020.

Supplementary Table S1. Summary of elevated measures of obesity and cardiovascular function.

	Female			Male		
	n	Cut off	%	n	Cut off	%
**BMI**	104	≥25	**40.0**	71	≥25	**43.8**
**BF%**	79	>31	**36.9**	43	>24	**31.9**
**WHR**	89	>0.85	**33.2**	96	>0.9	**56.5**
**HR**	16	>100	**8.2**	10	>100	**7.4**
**DP**	10	≥90	**4.6**	9	≥90	**6.6**
**SP**	18	≥140	**8.3**	28	≥140	**20.6**

Number (n) of female and male participants with value above the cut off for each measure of obesity (BMI, BF% & WHR) or cardiovascular function (HR, DP & SP) as percentage (%) of the cohort.
